# Oscillations by Minimal Bacterial Suicide Circuits Reveal Hidden Facets of Host-Circuit Physiology

**DOI:** 10.1371/journal.pone.0011909

**Published:** 2010-07-30

**Authors:** Philippe Marguet, Yu Tanouchi, Eric Spitz, Cameron Smith, Lingchong You

**Affiliations:** 1 Department of Biochemistry, Duke University Medical Center, Durham, North Carolina, United States of America; 2 Department of Biomedical Engineering, Duke University, Durham, North Carolina, United States of America; 3 Institute for Genome Sciences and Policy, Duke University Medical Center, Durham, North Carolina, United States of America; BMSI-A*STAR, Singapore

## Abstract

Synthetic biology seeks to enable programmed control of cellular behavior though engineered biological systems. These systems typically consist of synthetic circuits that function inside, and interact with, complex host cells possessing pre-existing metabolic and regulatory networks. Nevertheless, while designing systems, a simple well-defined interface between the synthetic gene circuit and the host is frequently assumed. We describe the generation of robust but unexpected oscillations in the densities of bacterium *Escherichia coli* populations by simple synthetic suicide circuits containing quorum components and a lysis gene. Contrary to design expectations, oscillations required neither the quorum sensing genes (*luxR* and *luxI*) nor known regulatory elements in the *P_luxI_* promoter. Instead, oscillations were likely due to density-dependent plasmid amplification that established a population-level negative feedback. A mathematical model based on this mechanism captures the key characteristics of oscillations, and model predictions regarding perturbations to plasmid amplification were experimentally validated. Our results underscore the importance of plasmid copy number and potential impact of “hidden interactions” on the behavior of engineered gene circuits - a major challenge for standardizing biological parts. As synthetic biology grows as a discipline, increasing value may be derived from tools that enable the assessment of parts in their final context.

## Introduction

Synthetic biology [1], [2], [3], [4], [5], [6] seeks to enable predictable engineering of cells and biological systems with altered or expanded function. Critical to this effort is the (re)design of information processing that establishes the timing and execution of cellular operations. At the molecular level, interpretation of particular internal and external stimuli is determined by the concentration and activity of cellular components (such as proteins, nucleic acids, and metabolites). Cellular responses, in turn, are executed by dynamically modulating these components in accordance with the cell's genetic program. The task of engineering synthetic systems therefore requires an understanding of not only the *system components*, but also their *interactions*, and the *control mechanisms* that adjust concentration and activity. In principle, a perfect understanding of these factors enables the development of models that accurately predict behavior for a proposed design. In reality, the scale and scope of cellular physiology, coupled with an imperfect understanding of the system and host components, make the construction of such models quite challenging. For this reason, simplified models that assume a well-defined interface between the circuit and host, while ignoring the background processes of host metabolism, are generally employed. If the predictions generated by the simple models deviate from the experimental implementation it indicates that the model insufficiently encompassed the critical components and interactions of the system. In this manner, the validity of common simplifying assumptions can partially addressed and refined during the design process.

In this work we present a genetic circuit (ePop) that causes oscillations in bacterial population density over time. The oscillations arise through the unanticipated interplay of growth conditions, the host cell, and one of the circuit's “background processes” – plasmid replication. Specifically, a density-dependent rise in plasmid copy number leads to an increase in gene dosage and concomitant increase in the expression of a plasmid-borne toxin gene. Toxin expression causes cell lysis and decreases the population density, allowing growth recovery, and generating oscillations as multiple cycles proceed. Although this mechanism was not intended during original circuit design, a refined mathematical model that incorporates plasmid copy number control captures the circuit dynamics, and predictions based on the model are validated by experimental results. Conditional plasmid amplification as a control mechanism for population density has not previously been described and evokes its potential for other applications. Furthermore, these results emphasize that copy number deserves increased attention when designing plasmid-based synthetic gene circuits.

One synthetic biology focus has been exhaustive documentation and standardization of individual biological parts [7]. While these efforts are valuable, circuits such as ePop exemplify the context-dependence of parts and devices and highlight the intrinsic difficulty in attempting to anticipate every possible circuit/host/condition interaction - even when using previously described and well-characterized parts, as is the case for ePop. Elucidating basic control mechanisms and improving tools that enable the assessment of parts in their final context is therefore important to the advancement of synthetic biology.

## Results

### The simple gene circuit ePop causes oscillations in cell density

**Figure 1:**
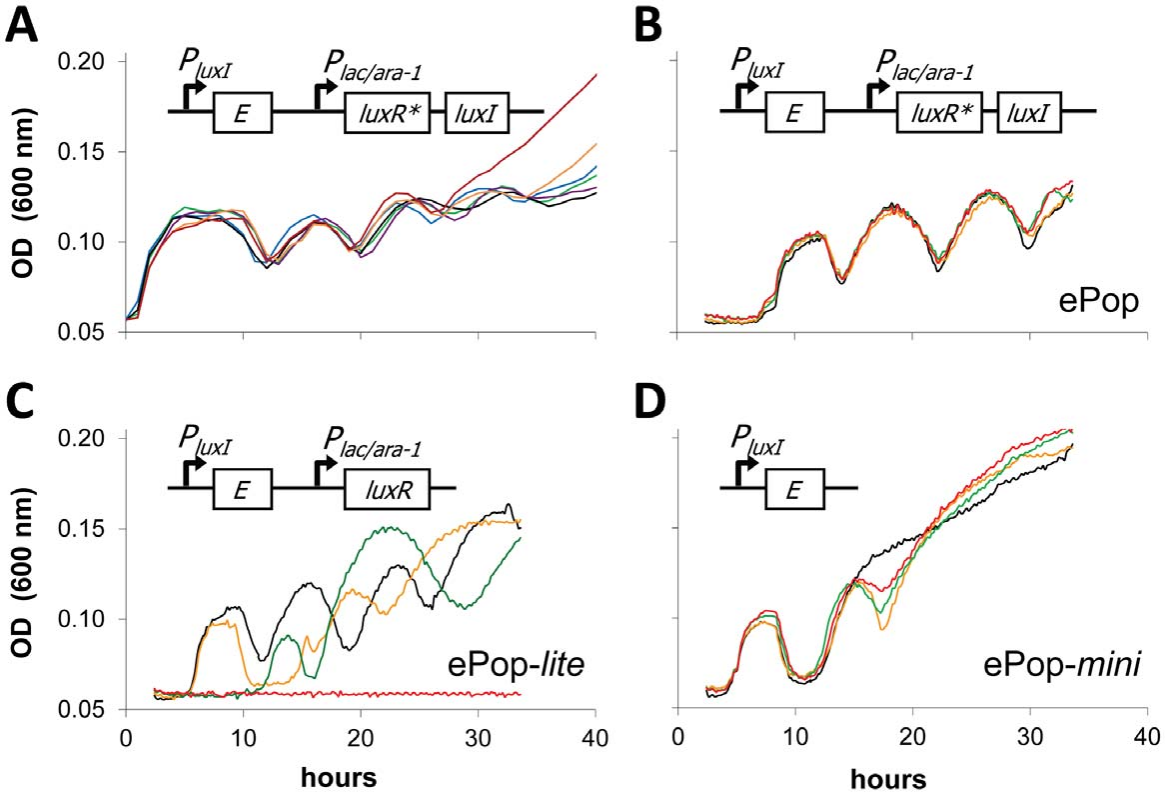
ePop dynamics in liquid culture. (A) MC4100z1 cells containing the ePop circuit (top) grown in liquid culture exhibited regular oscillations in cell density (bottom). Each trace represents a culture started from an individual colony. (B–D) Cultures treated with AHL: red (1000nM), green (100 nM), yellow (10nM), and black (0 nM). (B) Cells containing ePop oscillated independent of AHL concentrations. (C) Cells containing ePop-*lite* oscillated, but showed dose-dependent sensitivity to AHL (D) Cells containing ePop-*mini* oscillated independent of AHL concentrations (although only two cycles of lysis were observed here, other experiments with this plasmid showed up to four cycles).

The gene circuit (ePop) contains two modules (Figure 1A). The first module confers cell killing and consists of a lysis gene (*E*) from phage φX174 [8], [9] placed behind the *luxI* promoter (*P_luxI_*) from *Vibrio fischeri*, recently reclassified as *Aliivibrio fischeri* [10]. The *E* protein is an inhibitor of MraY, an enzyme that catalyzes the production of the first lipid intermediate in *E. coli* cell wall synthesis. Cells deficient in MraY activity lyse during septation, a process that requires newly synthesized cell wall [11]. The second module was intended to confer density sensing and consists of an inadvertently mutated *luxR* gene (luxR*) and an intact *luxI* gene under control of an isopropyl β-D-1-thiogalactopyranoside (IPTG) inducible promoter (*P_lac/ara-1_*). The *luxR* and *luxI* genes originate from *A. fischeri* and constitute a quorum sensing pair. LuxI produces a quorum signal, an acyl- homoserine lactone (AHL), from cellular precursors. LuxR is a transcription factor that responds to AHL and activates the *P_luxI_* promoter. In ePop, luxR* contains a frame-shift mutation that introduces an early stop codon and completely abolishes the luxR DNA-binding domain, as determined by homology to TraR [12]. ePop was implemented in a single plasmid that carries a chloramphenicol resistance gene and a ColE1 origin of replication that lacks the Rom/Rop protein.

When transformed into MC4100z1 cells, ePop generated multiple cycles of population oscillations during long-term batch cultures (Figure 1A). These oscillations were highly reproducible and showed only minor difference upon induction by IPTG (Figure S1B). MC4100z1 cells consistently displayed many cycles of oscillations, proceeding at times for five rounds of lysis. Oscillations were also observed in TOP10F', DH5α, BW25113 and MG1655. Colony forming unit (CFU) experiments (Figure S1C) showed that optical density reflected changes in viable cell density, although loss of viability appeared to precede lysis – consistent with previous characterization of E protein function. MC4100z1 cultures that did not carry ePop did not oscillate under these conditions (Figure S1D). The increasing baseline observed in optical density can be attributed to the accumulation of lysed ‘ghost’ cells. Solid phase measurements with BW25113 show synchronous growth arrest and lysis (Supplementary Movie S1). The MC4100z1 oscillations were among the most robust oscillations generated in a population of cells by synthetic gene circuits. Oscillations from past studies were less robust [13], [14], [15], operated among a sub-population of individual cells [13], [14], or required highly specialized devices to be observed [13], [14], [16], [17]. Generation of oscillations by synthetic circuits has recently been extended to mammalian cells [18].

To further characterize the oscillations, we investigated effects of culturing conditions specific to our growth assay, including media, temperature, and oxygenation. Complex media (Luria Broth) might have contributed to oscillations as shifts in preferred nutrient sources during growth could impact E protein expression. However, cells grown in defined M9 minimal media supplemented with glycerol exhibited oscillations similar to those grown in LB supplemented with glycerol (Figure S2A). Culturing temperature affects many parameters, such as growth rate and rates of cellular reactions – all of which might impact oscillations. Cultures grown at 33°C oscillated, but both the oscillation period and threshold OD were increased relative to cells grown at 37°C (Figure S2B). Because the majority of experiments were performed under mineral oil, and oxygen availability is a factor that affects luminescence in *V. fischeri*, anaerobic growth conditions might have contributed to oscillations. Aerobically grown cells underwent one or two rounds of lysis, but did not oscillate as well. These results indicate that oxygen availability contributed to but was insufficient to account for the observed oscillations.

### Oscillations do not depend on *luxRI* quorum sensing or promoter-level regulation

For oscillations to occur, the lysis phenotype conferred by the *E* gene must be triggered at elevated cell density. However, the *luxR* truncation and the low impact of IPTG induction suggested that the *luxR* and *luxI* genes were not responsible for density sensing. To test their role, we constructed two new versions of the ePop circuit. By design, both have the same *P_luxI_-E* module as ePop. ePop-*lite* contains functional *luxR* but no *luxI* (Figure 1C); ePop-*mini* contains neither *luxR* nor *luxI* (Figure 1D). Both ePop-*lite* and ePop-*mini* generated oscillations similar to those generated by ePop. Furthermore, exogenously added AHL did not affect oscillations by ePop (Figure 1B) or ePop-*mini* (Figure 1D) but suppressed growth of cells containing ePop-*lite*, in a dose-dependent manner (Figure 1C). These results confirmed that, unlike cells carrying ePop-*lite*, cells carrying ePop did not produce functional LuxR. More significantly, *luxR*, *luxI* and AHL-mediated cell-cell communication were shown dispensable for population oscillations. We conclude that LuxR* plays no role in oscillations given the truncation of the DNA-binding domain, insensitivity to AHL, and ability of circuits lacking it (ePop-lite) to oscillate.

**Figure 2:**
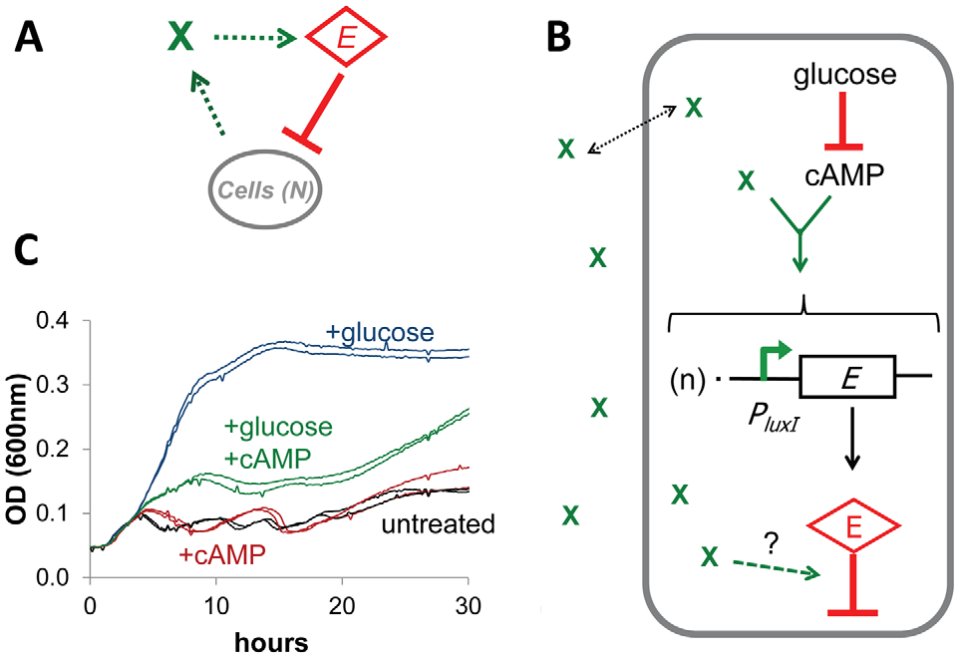
Possible sources of negative feedback. (**A**) Sustained population oscillations require negative feedback at the population level mediated by an unknown signal (X) and time delay. (**B**) Model for activation of the *P_luxI_* promoter integrating information from cAMP and X, where n represents the plasmid copy number per cell. The dashed line indicates the potential for post translational regulation, which we cannot rule out as a possibility. (**C**) Glucose and cAMP were added to LB and the impact on oscillations recorded. 1% glucose (blue) abolished oscillations; 5mM cAMP (red) slightly affected oscillation period; cultures containing 1% glucose and 5mM cAMP (green) reached an intermediate density and regained some oscillation. Cultures containing no exogenous glucose or cAMP are shown in black.

Oscillations require at least one negative feedback coupled with time delay (Figure 2A). In our circuits, time delay can be accounted for by the non-instantaneous rates of reactions affecting the level and function of the *E* protein, including transcription, translation, cell killing by the *E* protein, and *E* protein degradation. It is unclear, however, what control mechanism established feedback in ePop given the non-functional LuxR. Because the minimal oscillatory circuit was determined to be the *P_luxI_* promoter preceding the *E* gene, density-sensing must occur at either the level of *E* production or at the level of *E* protein activity (Figure 2B).

We hypothesized that regulation of mRNA production was a major cause of oscillations. This hypothesis predicts the existence of some signal X, whose level is a function of cell density, that either induces gene expression from the *P_luxI_* promoter or modulates gene dosage. Several candidates for X might be capable of inducing *E* gene transcription, such as the host cell's quorum sensing systems. Although *E. coli* do not possess a *luxI* homolog, their genome contains a *luxR* homolog, *sdiA*, which might activate the *P_luxI_* promoter. Two lines of evidence argued against this explanation. First, cells containing the ePop circuit did not respond to AHL (Figure 1C), which has been shown to bind SdiA [19] and activate gene expression. Second, oscillations were not significantly affected by indole (2mM), a stationary phase signal that has been reported to act through SdiA [20]. *E. coli* possesses a second quorum sensing system, the *luxS*-based AI-2 system [21], whose signal is derived from 4, 5-Dihydroxy-2, 3-pentanedione (DPD). Enzymatically produced DPD had a minor effect on oscillation dynamics, but did not inhibit growth in the manner that might be expected were it the feedback signal X (Figure S2C).

It has been shown that cyclic adenosine monophosphate (cAMP) and cAMP receptor protein (CRP) play a significant role in *V. fischeri* luminescence [22], [23]. In its natural genetic context, the *lux* promoter is located between the divergently expressed genes *luxR* and *luxI*. The literature suggests that cAMP-CRP activates the left-operon (*luxR*) and represses the right operon (*luxICDABE*). In ePop, the original CRP site is present and the *E* gene is located at the position of right operon. E expression and lysis was therefore predicted to be repressed by cAMP and stimulated glucose (which reduces cAMP concentrations). Paradoxically, glucose supplementation abolished oscillations and allowed elevated culture densities (Figure 2, blue lines). cAMP supplementation only slightly increased oscillation period and lysis density (Figure 2, red lines). When added concurrently with glucose, cAMP limited growth to intermediate densities, and some cycles of lysis were recovered (Figure 2, green lines); cAMP thus seemed necessary for oscillations to occur (Figure 2C). Nevertheless, exogenous cAMP neither prevented growth, nor increased lysis severity when added in the absence of glucose. These data suggest that cAMP is required for the manifestation of oscillations but is not the feedback signal X. Furthermore, these results were inconsistent with the previously reported promoter-level effects of cAMP on transcription in the *lux* operon.

**Figure 3:**
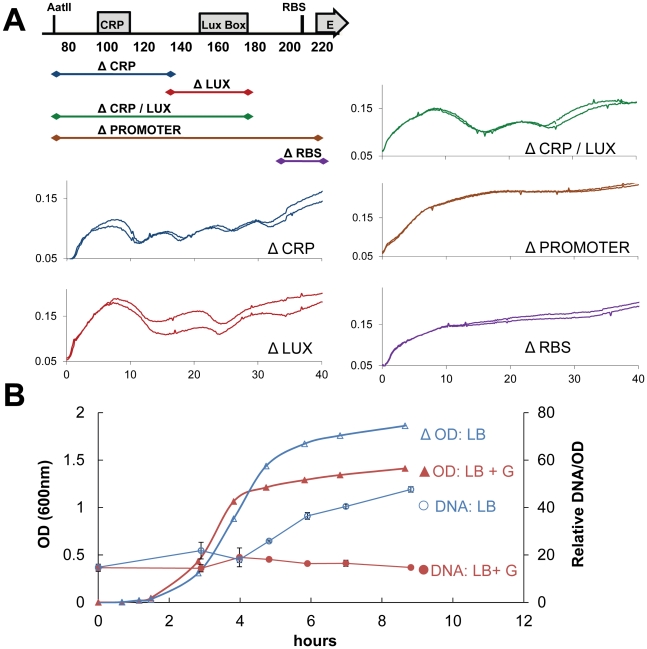
Plasmid amplification rather than promoter regulation may be the cause of oscillations. (**A**) Feedback is not at the promoter level. A promoter deletion series demonstrates that removal of the cAMP receptor site (ΔCRP), Lux box (ΔLUX), or both (ΔCRP/LUX) did not abolish oscillations. Deletion of the full promoter (ΔPROMOTER) did abolish oscillations, but this can be explained by deletion of the RNA polymerase binding site and ribosome binding site (ΔRBS). (**B**) OD (triangles) and miniprep yield (circles) from cells grown in LB (open symbols, blue) and LB + 0.2% glucose (closed symbols, red). Miniprep yield increases upon entry to stationary phase in the LB culture, but not in the glucose supplemented culture.

To address whether glucose and cAMP operated at the promoter level, as well as whether specific promoter regions required for oscillations could be isolated, a promoter deletion series was constructed (Figure 3A). ePop constructs that removed the *lux* box, cAMP site, or both maintained oscillations. Only those that deleted the core promoter site and the ribosome binding site abolished oscillations. These results demonstrate that transcriptional activation from upstream promoter elements, such as the *lux* box or CRP site, were not required for oscillations.

### Plasmid amplification can account for negative feedback

In the apparent absence of transcriptional regulation, we surmised that changes in gene dosage at higher cell density could cause oscillations. Modulation of DNA levels has been implicated in effects of iron deprivation on luminescence in *V. fischeri* as well as *E. coli* transformed with the *lux* operon from *V. fischeri* [22]. To investigate plasmid amplification, MC4100z1 cells carrying plasmid pNewTet.E, which is a backbone vector of ePop containing a resistance marker and origin of replication identical to that of ePop, were grown in the presence and absence of glucose. Plasmid content at various time points was measured by the miniprep yield of culture samples resuspended to similar ODs. Final values were normalized to OD readings to account for slight difference in the density of resuspended cultures. pNewTet.E was used rather than ePop because cells in the process of lysing are fragile and cannot withstand the miniprep protocol. Given the identical plasmid backbone, plasmid amplification should impact copy number similarly. This allows pNewTet.E to serve as proxy for ePop for the purposes of addressing copy number as a function of culture phase under different media conditions. Miniprep yield/OD was found to increase upon transition to stationary phase in the LB grown cultures, but not those supplemented with glucose (Figure 3B). Together with the existing understanding of ColE1 plasmid copy number control, this data suggested a mechanism for both density sensing and inhibition of oscillations by glucose – plasmid amplification.

### ColE1 replication

**Figure 4:**
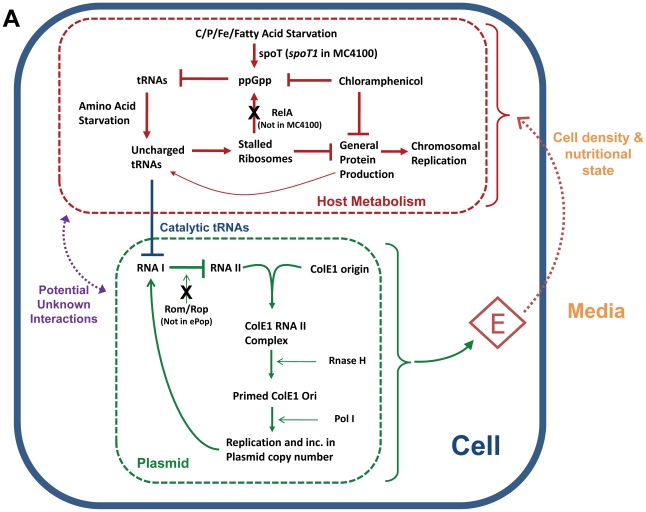
Modeling plasmid amplification, lysis, and oscillations. The diagram shows stringent control, plasmid replication, and a possible mechanistic link. The host stringent response prepares *E. coli* cells for prolonged periods of nutritional limitation through the control of ppGpp levels [56], [57]. Although the ppGpp response is multifaceted, for simplicity, only the regulation of tRNAs is shown. In wild-type cells ppGpp is either produced by RelA as a consequence of uncharged tRNAs resulting from amino acid starvation, or by SpoT in response to other nutritional stresses. However, because MC4100 cells are relaxed (*relA1* allele) ppGpp is not produced in response to amino acid starvation and uncharged tRNA levels can accumulate to a greater degree. Uncharged tRNAs have been shown to degrade RNA I, the negative regulator of plasmid replication, *in vitro*, and lead to plasmid amplification when overexpressed *in vivo* [32]. Indeed, relaxed hosts experience ColE1 plasmid amplification when starved for amino acids. Our observations on ePop are consistent with a model where uncharged tRNAs accumulate and plasmid is amplified at high cell density and nutrient limitation. Low nutrient goes unacknowledged by the cell because RelA is not present to sense uncharged tRNAs and chloramphenicol is present to inhibit ppGpp accumulation. Plasmid amplification leads to increased E expression, cell lysis, decreased population density, and subsequent release of nutrient limitation. Although this model can account for the observations, we cannot exclude the possibility that other interactions exist to provide alternate or additional linkage between host metabolism and plasmid replication.

Regulation of replication in ColE1-type plasmids has been well characterized, *for reviews see* [24], [25]. Briefly, the origin codes for two constitutively expressed regulatory RNAs. One is an RNA primer (RNA II) that initiates replication after recognition and cleavage by RNase H. The other (RNA I) is short-lived, more highly expressed, antisense RNA that associates with RNA II and inhibits processing by RNase H, and therefore replication. In ColE1-type origins that lack the Rom/Rop protein (like ePop), basal copy number is increased because Rom/Rop stabilizes the RNA I/RNA II interaction. This feedback provides a means of copy number control because levels of RNA I increase with copy number and serve to inhibit further replication (Figure 4). A consequence of this mechanism is that copy number is very dependent on factors that affect RNA I production and degradation.

Relevant to the ePop dynamics, studies have identified several environmental factors that increase ColE1 copy number. Chloramphenicol causes the arrest of protein synthesis and chromosomal replication while stimulating replication from the ColE1 origin resulting in drastic plasmid amplification [26]. Amino acid starvation can result in amplification similar to chloramphenicol and was found to be particularly pronounced in relaxed strains [27] invoking a role for the stringent response mediators (p)ppGpp, relA, and spoT. Also, growth rate is inversely related to copy number across cells strains as well as by altering media composition for a given cell strain [28], [29]. This relationship is observed in both *relA*+ and *relA* strains (Lin-Chao et al. 1986) and is somewhat enhanced for plasmid lacking the Rom/Rop protein [30]. Finally, IPTG can lead to increased copy number of ColE1 plasmids, but only when protein expression caused a decreased growth rate and drop in ppGpp levels [31].

Details on a mechanistic link between environmental factors and ColE1 replication have recently emerged and suggest that uncharged tRNAs directly catalyze the degradation of RNA I thereby dis-inhibiting replication [32]. Accumulation of uncharged tRNAs can be mitigated by the stringent response and ppGpp accumulation, which is perturbed in MC4100z1 [33], and prevented by chloramphenicol respectively [34], [35]. For a brief discussion of the interplay between ColE1 replication, stringent control, and plasmid amplification in MC4100z1, see Figure 4.

### Construction of a mathematic model that captures ePop dynamics

The complexity and number of inputs inherent to the both the stringent response and ColE1-type plasmid regulation (only partially encompassed in Figure 4) challenge the construction and parameterization of a comprehensive and accurate mathematical model. To this end, we have constructed a drastically simplified model to capture the key aspects of observed dynamics. The purpose of our model is not to intricately simulate ColE1 copy number control or its interaction with metabolism, and more detailed models of these types have been built [36], [37], [38], [39], [40]. Rather the model provides a simplified framework to interrogate effects of the perturbations on plasmid copy number amplification with regards to ePop oscillations.

**Figure 5:**
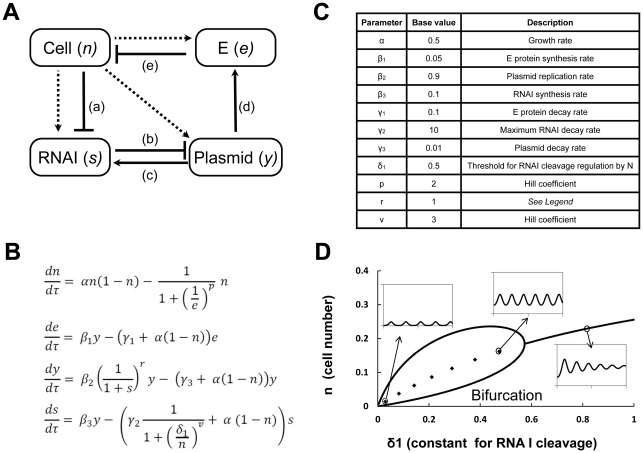
A simplified model for ePop function. (**A**) Solid lines indicate positive and negative regulation. Dashed lines represent the effect of cell growth on component dilution. a. Increasing cells density causes RNA I degradation (possibly through uncharged tRNAs. See Figure 4 for more details). b. RNA I inhibits plasmid replication (through its interaction with RNA II). c. RNA I is produced from the ePop plasmid; elevated plasmid levels increase RNA I production. d. E protein is produced from ePop plasmid by basal expression from the *luxI* promoter in the absence of functional LuxR. Elevated plasmid levels increase E protein production. e. E protein decreases cell density by blocking cell-wall synthesis and lysing cells. (B) Dimensionless ODE model of the circuit. Changes in cell density (*n*) are modeled as logistic growth with an intrinsic growth rate, α. We assume that killing of cells by the E protein is cooperative and describe it using a Hill-type function (Hill coefficient, *p*). We note that cooperatively of E protein-mediated killing is not required for generating oscillations. The E protein is produced from a plasmid (*y*) with a rate β_1_ and degraded with a rate γ_1_; both processes follow first-order kinetics with regards to the amount of plasmid and E protein, respectively. Plasmid replication is inhibited by RNA I (s), and replication inhibition follows a power of hyperbolic function where *r* is the effective number of reaction steps in the inhibitory scheme [1]. β_2_ sets the maximum plasmid replication rate and γ_3_ the intrinsic decay rate. RNA I is produced from the plasmid with a rate β_3_ whereas its degradation rate is dependent on the cell density. Degradation of RNA I is described by a Hill-type function (Hill coefficient, *v*) to account for possible cooperativity. E protein, plasmid and RNA II are subject to dilution with cell growth. (**C**) The base parameter set that can generate sustained oscillations. Rate coefficients are normalized to a maximum killing rate (i.e. the maximum cell killing rate by E protein is 1). Biologically relevant parameter values have been chosen to illustrate the basic dynamics. E protein production rate β_1_ is set to be small to reflect leaky expression. Plasmid decay rate γ_3_ is set small to reflect the stability of plasmid molecules, and under oscillatory conditions plasmid dilution dominates. (**D**) Bifurcation diagram showing a region of sustained oscillations over varying ‘half-maximal constant for RNAI cleavage’ (δ_1_). Insets show simulated time courses of cell density for three δ_1_ values. Damped oscillations can be generated outside the bifurcation region.

We propose the following: *E. coli* growth proceeds until culture density is sufficiently high. At high cell density, RNA I levels and division rate decrease, causing plasmid amplification, leading to increased basal production of E. Lysis of the majority of the cells results in nutrient release and loss of cell density signals allowing growth recovery and a subsequent cycle of growth and lysis. A schematic of these major reactions is shown (Figure 5A). A system of ordinary differential equations describes key reactions for the four basic model components: cell growth and killing, E protein accumulation, plasmid amplification, and modulation of RNA I levels (Figure 5B). By broadly considering the impact of cell density on RNA I, the model is equivalently valid whether uncharged tRNAs provides the feedback or some other molecular mechanisms are involved in coupling cell density to plasmid amplification.

### Perturbations that modulate copy number impact oscillations

Parameterized appropriately (Figure 5C), the model can generate sustained or damped oscillations in the absence of promoter-level or post-transcriptional regulation. It cannot be determined from batch culture experiments whether the oscillations caused by ePop are damped or sustained because conditions change as nutrients are depleted. However, the model can indicate parametric space that promotes sustained oscillations. A key parameter that determines the strength of feedback between cell density and copy number control is δ_1_, the half-maximal constant for RNAI cleavage by cell density. Bifurcation analysis indicates that oscillations become sustained for when δ_1_ is sufficiently small (Figure 5D).

**Figure 6:**
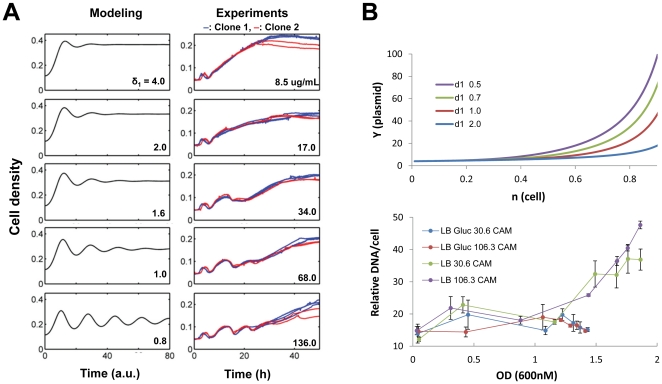
Model predictions and experimental responses to system perturbations. (**A**) Model predictions of increasing δ_1_ outside the bifurcation region on oscillations (left) match the result of decreasing chloramphenicol concentration (right), providing further support for the plasmid amplification mechanism. All chloramphenicol concentrations tested completely inhibited the growth of MC4100z1 cells without ePop and are therefore sufficient to prevent the growth of plasmid free segregates. (**B**) (**top**) Simulation of plasmid levels as a function of cell density in the absence of E protein (β_1_ set to 0). Increasing δ_1_ values result in depressed plasmid amplification. (**bottom**) Experimental data of plasmid amplification (plotted as DNA/cell as a function of OD) demonstrate adding glucose or lowing chloramphenicol concentrations have the apparent effect of increasing δ_1_. Glucose when present was used at 0.2% and chloramphenicol concentrations were 30.6µg/mL or 106 µg/mL. Coloring of traces is meant to demonstrate the trend and should not imply a direct quantitative agreement of specific model values with specific culturing conditions. The two traces at 106 µg/mL are from the same data as Figure 2B.

Given the established link between chloramphenicol and plasmid amplification (Figure 4), we hypothesized that if ePop oscillated though a plasmid amplification mechanism, increasing chloramphenicol should mirror the effect of decreasing δ_1_. According to our model, decreasing δ_1_ would lead to an increasing number of cycles of damped oscillations (Figure 6A, left column) or sustained oscillations (corresponding to an infinite number of cycles, Figure 5D). Experimentally, we indeed observed an increasing number of oscillations increasing chloramphenicol concentrations (Figure 6A, right column), consistent with the model prediction.

Similarly, the model provides an interpretation for the effects of glucose. If E synthesis rate (β_1_) is set to zero, lysis does not occur and the model reduces to a simplified treatment ColE1 copy number control. Increasing δ_1_ in this context minimizes the plasmid amplification that occurs as stationary phase is reached and mirrors the impact of glucose on the accumulation of the ePop backbone pNewTet.E (Figure 6B). If we consider the impact of glucose on ePop dynamics as being mediated through an increase in δ_1_ then the observation that glucose abolishes oscillations (Figure 2C) is not surprising. On a molecular level it is more difficult to attribute the precise role played by glucose, which could exert an impact through growth rate, ppGpp production/degradation by SpoT, overall energetic state or any number of host pathways under catabolite control.

## Discussion

**Figure 7:**
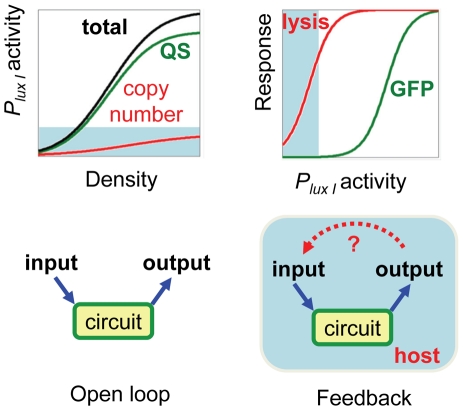
Gate matching and unexpected feedbacks. (**top**) Total *P_luxI_* activity is the combination of plasmid amplification and quorum sensing. In ePop, defective LuxR prevents the contribution from quorum sensing - leaving only that of plasmid amplification. Low *P_luxI_* activity is sufficient to cause lysis, due to the extreme toxicity of the E gene. A more typical reporter used for promoter characterization (such as GFP) may be undetectable at this level, causing the effects of plasmid amplification to be missed. (**bottom**) A gene circuit can be designed as an open loop to process a series of inputs into defined outputs. When circuits are placed into host cells, however, hidden interactions between circuit and cellular components can introduce feedback that significantly impacts circuit dynamics. In ePop, the interaction between cell density and plasmid amplification is an unanticipated feedback that allows the circuit output (cell density) to serve as an input by modulating gene dosage.

Given the frequent application of the quorum sensing in gene circuits [41], [42], [43], [44], [45], [46], [47], [48], it is perhaps surprising that quorum sensing-like behavior resulting from plasmid amplification has not been described or encountered in synthetic systems until now. One possible explanation is due to differences in the threshold at which downstream genes become effective, underscoring the importance of matching the dynamic range of input/output elements in a circuit (Figure 7). Cell density induced increases in mRNA production should result in comparable expression of either the E protein or a typical reporter protein, such as the green fluorescent protein (GFP). Whereas low levels of the *E* protein can cause loss of viability [49], at the same level, the reporter protein may be below the detection threshold of standard methods [7]. Our results also suggest that plasmid amplification is most pronounced when the ColE1 origin and chloramphenicol are used in tandem. For this reason the chloramphenicol resistance marker could be considered partially incompatible with the ColE1 origin when minimal plasmid copy number change is desired. In addition, it appears that culturing conditions and cell strain have a significant impact on oscillations. Perhaps the confluence of needed conditions for significant plasmid amplification was not met in the past. In this regards, ePop itself can be valuable as a probe of cell physiology to interrogate what culturing and genetic conditions must be met for plasmid amplification. For example, observations on the impact of glucose on ePop oscillations motivated the experiments demonstrating that glucose inhibited plasmid amplification (Figure 3B). As a probe, ePop has the advantage of a simple observable (OD), high sensitivity, and rich information from complex dynamics. A better understanding of the factors that influence ColE1 control in a particular genetic and environmental context will enable the exploitation of plasmid amplification as a feature in future circuits.

The ease with which plasmids are isolated, genetically engineered, and re-introduced has led to their near ubiquitous use in synthetic biology, despite the potential consequences of variable copy number. Theoretical work has shown that even small changes to copy number values can result in significant non-linear effects on simple network motifs [50]. Nevertheless, plasmid copy number is often ignored under the assumption that any variation (basal or dynamic) will not have a large impact on the overall performance of the genetic device. In the case of ePop, this assumption was violated as plasmid amplification appears to be the cause of population oscillations. Placement of a circuit subsystem like copy control in a black box while ignoring molecular details is not faulty *per se*, and often necessary in order to initiate the engineering of large-scale systems [51]. However, it is most appropriate when the input/output relationship of the boxed subsystem is well defined. Unfortunately, no equation exists that takes any combination of strain genome, media composition, temperature, and chloramphenicol concentration into account while accurately returning copy number as a function of cell density.

ePop therefore underscores a fundamental challenge in standardizing cellular parts for synthetic biology [4], [52]. The value of information from standardized parts is critically dependent how closely characterization conditions mirror implementation conditions. When choosing parts for engineering a circuit, literature data are the primary resource, but such data are often available for the parts only in their natural context or a narrow range of characterization conditions. Because biological parts are influenced by, and exert their influence through, interactions with other parts and their host cell, their behavior likely changes with their context. For example, even identical parts and network motifs can behave differently depending on their physical DNA configuration [53]. Circuit-host interactions (Figure 7) can drastically influence dynamics as evidenced by this work and the recent example of circuit-induced growth retardation leading to bistability [54]. Furthermore, it is impossible to assume that all of a part's functions and interactions have been determined, even for well-characterized systems. While the definition of “standard” biological parts and the concept of parts abstraction and hierarchical composition can often simplify circuit design and analysis, these strategies can drastically underestimate the potential complexity of circuit dynamics. The likelihood that circuit behavior will deviate from predictions derived from characterization information scales non-linearly with the size and complexity of circuit as each new part can have unexpected interactions with every other circuit component and the host strain.

These issues call for greater emphasis on the development of methods to produce and monitor systems using parts for which data are incomplete, and whose behavior may change when placed in a new setting. Every circuit whose real-world behavior varies dramatically from our best predictions represents an opportunity to better understand the components, interactions, and control mechanisms of both the system and the host. The advantages of a standard parts-based approach should not obscure the fundamental biological insight that can be gained from a more holistic analysis of synthetic systems and their emergent properties – especially when those systems “fail” to behave as anticipated.

## Materials and Methods

### Strains, growth conditions, chemicals and media

MC4100Z1 cells were the gift of M Elowitz. BW25113 and MG1655 were obtained from the *E. coli* stock center. Unless otherwise noted Luria Broth (LB) buffered with 100mM MOPS (pH = 7.0) was used for cell growth. 3oxoC6 homoserine lactone (AHL) was synthesized by the Duke Small Molecule Synthesis Facility. DPD was produced using *pfs* and *luxS* genes cloned from MG1655, expressed in BL21 (DE3) cells using pET-21 and purified using an immobilized metal affinity chromatography column (GE Healthcare). The enzymatic reaction was performed as previously described [55] and 5,5′-Dithio-bis(2-nitrobenzoic acid) (DTNB, Ellman's reagent) was used to calculate yield on the basis of homocysteine released. All other chemicals were purchased from Sigma-Aldrich.

### Liquid culture monitoring

Cells were grown in 48 well Tissue Culture Plates (Falcon 353078, BD Labware) inside a Perkin-Elmer VICTOR3 plate reader heated to 37°C unless otherwise noted. Wells containing 500µL fresh media and the appropriate chemicals were inoculated with 50µL of a starter culture (grown aerobically in a 5mL culture tube for 2–4h from glycerol stocks or fresh colonies). 250µL mineral oil was layered above each well to prevent evaporation over the course of the experiment. Care was taken to ensure starter cultures did not reach densities high enough to induce lysis. Prior to each measurement, plates were shaken for 5 sec in an orbital pattern. OD at 600nm was measured every 15 min. Data presented are the raw OD values that result from 500uL culture in the plates used. They have not been modified to account for path length.

### Plasmids

ePop (ColE1 origin, chloramphenicol^R^) was constructed using the *lux box* region (140 bp upstream of *luxI* in *V. fischeri*) from p*lux*GFPuv [43] and *E* gene coding sequence from φX174 (NEB). Each region was PCR-amplified and then joined together in an overlap PCR reaction. The ‘*lux* box-*E* gene’ fragment was inserted into the AatII site of host vector pLuxRI2 [44]. ePop-*mini* was constructed by inserting the *lux* box-*E* gene fragment (AatII digest of ePop) into pLuxR2 [44] at the AatII site. ePop-*lite* was constructed by digesting ePop with HindIII (deleting most of *luxR* and all of *luxI*) and re-ligating the larger fragment from an agarose gel purification. Promoter deletion mutants were generated using divergent primers flanking the deletion of interest. Each primer introduced a terminal NheI site. Digestion and intramolecular ligation generated plasmids where the region targeted for deletion was replaced by a 6 base pair NheI “scar”. pNewTet.E was generated by introducing a 40bp oligo with NdeI and NheI sites into the AatII site of pProTet.E (Clontech).

### Plasmid content determination

A starter culture of MC4100z1 cells carrying the pNewTet.E plasmid was grown until it had entered early logistic growth (OD = 0.28). 750µL of starter culture was added to 2L of LB or LB+glucose (0.2% final concentration) in 6L non-baffled flasks and grown at 37C and 180rpm. Chloramphenicol was added to the media to a final concentration of 30.6 µg/mL or 106µg/mL. An equivalent OD of cells was removed at various time points using a previously determined calibration curve to account of the non-linearity of OD measurements. Cultures were centrifuged for 10min at 5000g and re-suspended in 2mL Tris-buffered Saline. 240µL of the resuspended culture was added to 2mL LB and OD was recorded. Resuspended OD values were all within 20% of one another, enabling linear normalization. The remaining re-suspended culture was used to perform triplicate 500µL minipreps using the Zyppy miniprep kit (Zymo Research). Miniprep yield was measured on a ND-1000 UV spectrophotometer (Nanodrop).

## Supporting Information

Figure S1 OD monitoring of MC4100z1 cells carrying the ePop circuit. Cells were grown in the absence **(A)** or presence **(B)** of 1mM IPTG and OD (600nm) was measured (every hour in this case) in a plate reader. 1mM IPTG did not drastically change the nature of the oscillations but did affect the synchronization across different colonies and starter cultures (different colored traces) **(C)** Colony forming unit (CFU) experiments were performed every three hours and show that viable cell density correlated with OD. **(D)** MC4100z1 cells that do not carry any plasmids (red) do not show the oscillations exhibited by MC4100z1 cells that carry the ePop plasmid (green) demonstrating that strain and culturing conditions were insufficient to produce oscillations.
(0.48 MB TIF)

Figure S2 Effect of changing culturing conditions. **(A)** Cells grown in buffered LB supplemented with glycerol (top) showed similar oscillations to cells grown in M9 minimal media supplemented with glycerol (bottom). Different colored traces represent individual colonies. Oscillations are therefore not due to some unknown component in complex media or shifts in preferred media source. **(B)** Cells grown at 33C (black) exhibit an elevated lysis density and longer period than those grown at 37C (red). **(C)** Cultures treaded with DPD exhibited one of two phenotypes in response. Cells either oscillated with a similar period but recovered more quickly from an initial round of lysis **(top)** or had oscillation period significantly increased by DPD **(bottom)**. The differences between the two types of colonies, presumably of genetic origin, have not been determined. DPD and AI-2 did not appear to be the feedback signal X, however, because neither prevented growth or caused increased lysis.
(0.57 MB TIF)

Movie S1 Solid phase growth monitoring of BW25113 cells carrying the ePop plasmid. Microscope slides were prepared by allowing warm LB agarose (1%) containing the appropriate antibiotics and inducers (1mM IPTG, 100ng/uL aTc) to be drawn under a suspended cover slip. After cooling, the coverslip was removed and the agarose pad cut to size with a clean razor blade. 1 µL low density starter culture (BW25113, ePop) was placed on the agarose and a fresh coverslip was applied. Slides were sealed with mineral oil and nailpolish to prevent evaporation. Cells were placed on a Leica inverted microscope in an environmentally controlled enclosure set to 37°C. After 1–2 hours, a region for monitoring was selected on the basis of exhibiting cell division. Images were captured using a Hammamatsu 1384 ORCA-ERA camera at 4 frames per minute overnight using autofocus feature to maintain the focal plane.
(4.17 MB MP4)
