# Supplementary figures and images for: Oscillations by Minimal Bacterial Suicide Circuits Reveal Hidden Facets of Host-Circuit Physiology
Source: PLoS One. 2010 Jul 30;5(7):e11909. doi: 10.1371/journal.pone.0011909 (PMC2912849; doi:10.1371/journal.pone.0011909)

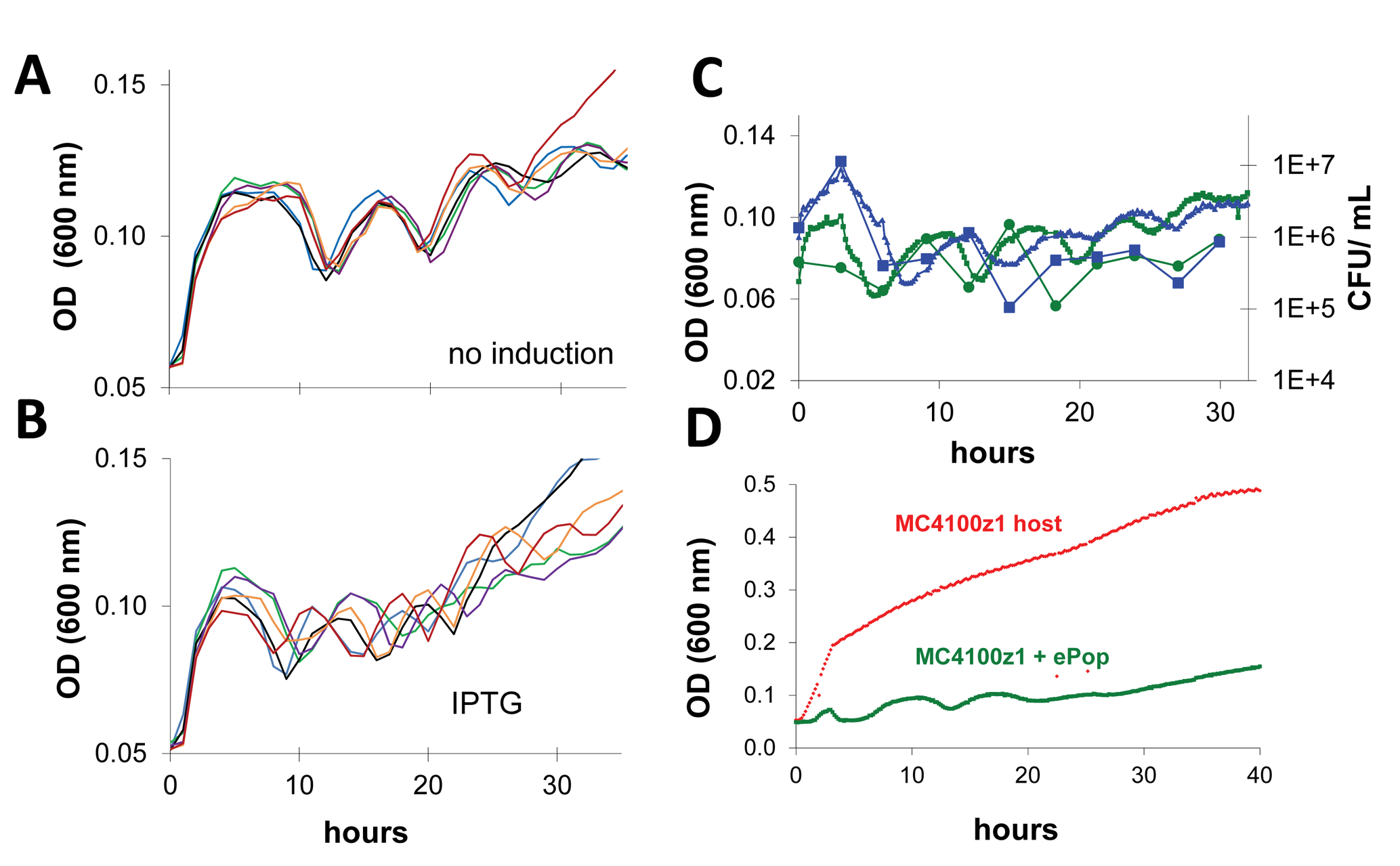

Supplement: Figure S1 — OD monitoring of MC4100z1 cells carrying the ePop circuit. Cells were grown in the absence (A) or presence (B) of 1mM IPTG and OD (600nm) was measured (every hour in this case) in a plate reader. 1mM IPTG did not drastically change the nature of the oscillations but did affect the synchronization across different colonies and starter cultures (different colored traces) (C) Colony forming unit (CFU) experiments were performed every three hours and show that viable cell density correlated with OD. (D) MC4100z1 cells that do not carry any plasmids (red) do not show the oscillations exhibited by MC4100z1 cells that carry the ePop plasmid (green) demonstrating that strain and culturing conditions were insufficient to produce oscillations. (0.48 MB TIF) [file pone.0011909.s001.tif]

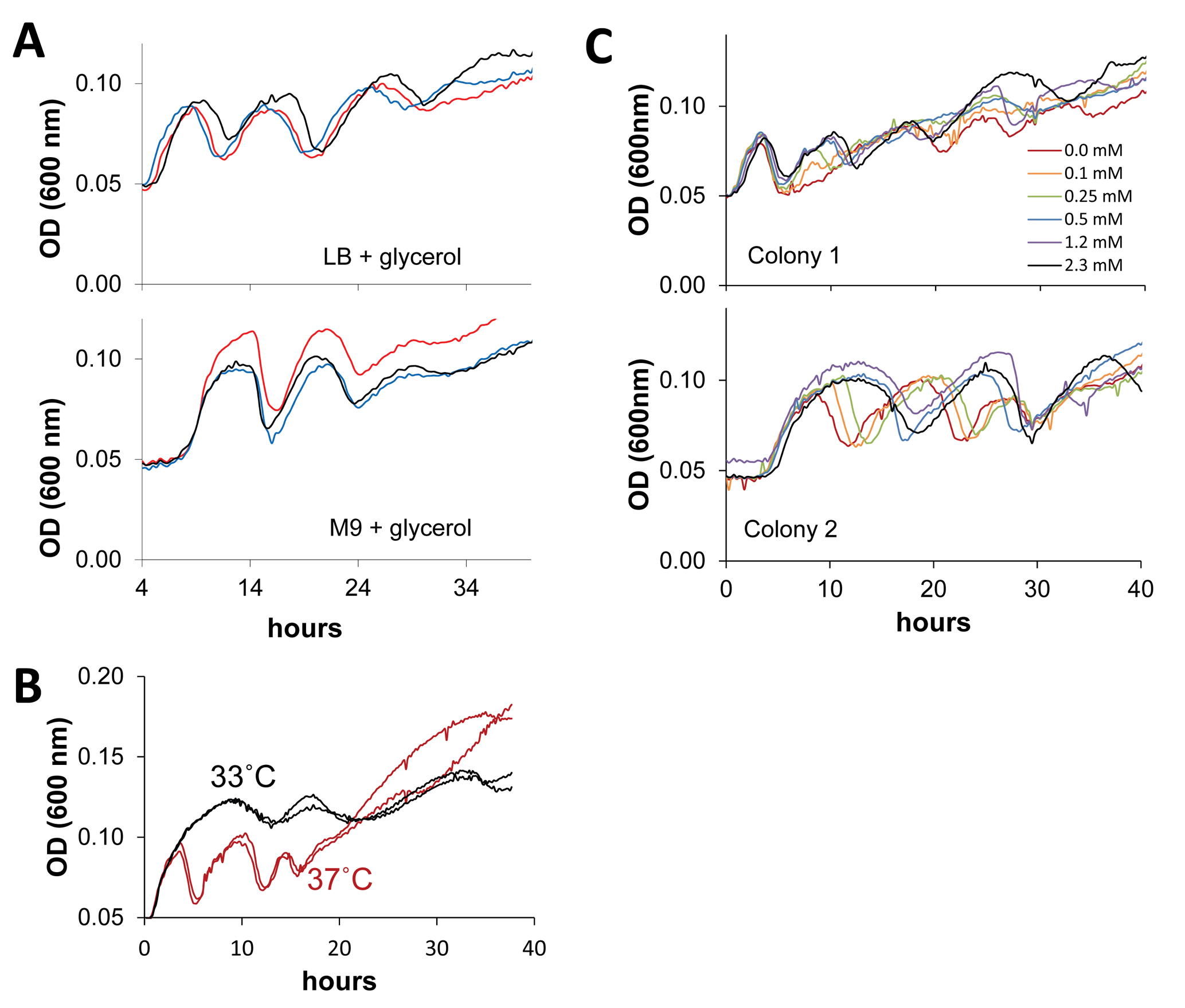

Supplement: Figure S2 — Effect of changing culturing conditions. (A) Cells grown in buffered LB supplemented with glycerol (top) showed similar oscillations to cells grown in M9 minimal media supplemented with glycerol (bottom). Different colored traces represent individual colonies. Oscillations are therefore not due to some unknown component in complex media or shifts in preferred media source. (B) Cells grown at 33C (black) exhibit an elevated lysis density and longer period than those grown at 37C (red). (C) Cultures treaded with DPD exhibited one of two phenotypes in response. Cells either oscillated with a similar period but recovered more quickly from an initial round of lysis (top) or had oscillation period significantly increased by DPD (bottom). The differences between the two types of colonies, presumably of genetic origin, have not been determined. DPD and AI-2 did not appear to be the feedback signal X, however, because neither prevented growth or caused increased lysis. (0.57 MB TIF) [file pone.0011909.s002.tif]
